# Connections between diabetes, depression, and purpose in life in older adults

**DOI:** 10.1093/geroni/igaf122.2260

**Published:** 2025-12-31

**Authors:** Jonathan Sundby, Tiana Broen, Leilani Feliciano

**Affiliations:** University of Colorado Colorado Springs, Colorado Springs, Colorado, United States; University of Colorado Colorado Springs, Colorado Springs, Colorado, United States; University of Colorado Colorado Springs Department of Psychology, Colorado Springs, Colorado, United States

## Abstract

Purpose in life is defined as the belief that one’s life has goals, direction, and meaning. In older adulthood, purpose in life is an important component of well-being and previous literature has found it to be associated with more “successful aging,” including inner strength and social integration (AshaRani et al., 2022). Our project aimed to examine whether the experience of living with diabetes impacts purpose in life in older adulthood. Diabetes is a chronic disease that requires regular monitoring of blood sugar levels, as well as lifestyle changes (dietary, exercise, medication), to prevent serious medical complications or fatal consequences. We hypothesized that the experience of living with diabetes erodes a person’s purpose in life, partially explained through the mechanism of increased risk of depression (Cho et al., 2024). Data were analyzed from older adults (65+ years; n = 1358) in the third wave of the Midlife in the United States (MIDUS) dataset. A series of linear regressions indicated that the presence of diabetes predicted lower purpose in life [β = -.098, p < .001], as well as a higher likelihood of experiencing depression [B=.372, p = .025]. Running both variables together in a hierarchical linear regression led to a smaller, but still significant, role for diabetes within the model [β = -.086, p = .001]. These results emphasize the connections between the presence of chronic disease and purpose in life and highlight that effective treatment of physical conditions alongside their mental health comorbidities could help increase the well-being of older adults.

